# Co-Occurrence of Multidrug Resistant Klebsiella pneumoniae Pathogenic Clones of Human Relevance in an Equine Pneumonia Case

**DOI:** 10.1128/spectrum.02158-21

**Published:** 2022-05-17

**Authors:** Carola Venturini, Bethany Bowring, Sally R. Partridge, Nouri L. Ben Zakour, Alicia Fajardo-Lubian, Ariana Lopez Ayala, Jilong Qin, Makrina Totsika, Gaby van Galen, Jacqueline Norris, Jonathan Iredell

**Affiliations:** a Centre for Infectious Diseases and Microbiology, Westmead Institute for Medical Research, Westmead, New South Wales, Australia; b Sydney School of Veterinary Science, University of Sydney, Sydney, New South Wales, Australia; c Sydney School of Medicine, University of Sydney, Sydney, New South Wales, Australia; d Westmead Hospital, Western Sydney Local Health District (WSLHD), Westmead, New South Wales, Australia; e Centre for Immunology and Infection Control, School of Biomedical Sciences, Queensland University of Technology, Brisbane, Queensland, Australia; Nanjing Agricultural University

**Keywords:** *Klebsiella pneumoniae*, ST307, multidrug resistance, bacteriophages, plasmids, pathogenic clones

## Abstract

The global epidemiology of multidrug resistant Klebsiella pneumoniae, a serious threat to both animal and human health, is dominated by the spread of pathogenic clones, each separately evolving via acquisition of transferable antibiotic resistance or niche-specific virulence determinants. In horses, K. pneumoniae infection can lead to severe respiratory illness. Here, we characterized multiple isolates recovered from bronchial aspirates of a mare with pneumonia refractory to antibiotics. First, we used a combination of standard microbiology, bacteriophage cross-susceptibility and antibiotic resistance testing to profile the infecting K. pneumoniae population. The genomes of isolates with distinct fingerprints (pulsed-field gel electrophoresis) and unique combined bacteriophage/antibiotic profiles were then further analyzed using whole-genome sequencing. Adhesion to human epithelial cells and biofilm production were also measured as virulence indicators. Although it is commonly expected for one clone to dominate an infection episode, we identified five coexisting multidrug resistant K. pneumoniae sharing the same niche. One was a novel sequence type (ST4656), while the other four were all members of emerging human pathogenic clonal groups (ST307, ST628, ST893 and ST392). These isolates did not display significant differences from one another in terms of virulence or resistance and differed only in plasmid content from isolates implicated in severe human infections, with equal potential to prolong duration and severity of infection when sharing the same niche. This study highlights the importance of more precise surveillance and detection measures to uncover bacterial heterogeneity, reminding us that the “single clone” concept is not an absolute in invasive bacterial infections.

**IMPORTANCE** Multidrug resistant Klebsiella pneumoniae are agents of life-threatening infections in animals and humans, with several multidrug resistant clones causing outbreaks of disease worldwide. It is generally accepted that only one clone will be dominant in an infection episode. In this study, we investigated K. pneumoniae isolates from a horse with severe pneumonia and demonstrated co-occurrence of multiple sequence types previously identified as emerging human pathogens. The equine isolates are not significantly different from one another in terms of virulence or resistance, with equal potential to prolong duration and severity of infection, and are indistinguishable from isolates recovered from humans, except for plasmid content. Our study highlights how the “one dominant clone” concept is not an absolute in severe infection, illustrating the need for improved diagnostics to track heterogeneity of infection, and reinforces the importance of cross-monitoring of environmental and human reservoirs of multidrug resistant pathogens.

## INTRODUCTION

Klebsiella pneumoniae, a ubiquitous bacterial species, is a common resident of the healthy mammalian gut, but also a leading cause of opportunistic, often severe, extra-intestinal infections, including sepsis and meningitis, which are routinely treated with antibiotics (1). The rise in antimicrobial resistance in this species has therefore become a primary medical and veterinary concern (2–5). Among multidrug resistant (MDR) K. pneumoniae, strains producing extended spectrum β-lactamases and carbapenemases have been classified as an urgent threat by health agencies worldwide (2, 3). In animals, MDR K. pneumoniae infection can lead to life-threatening conditions, such as severe mastitis in cows and hemorrhagic pneumonia in horses, increasing treatment costs, particularly when refractory to commonly used antibiotics (e.g., gentamicin or trimethoprim) (4, 6, 7).

The evolution of K. pneumoniae pathotypes is characterized by diversification of closely related lineages, leading to separation into distinct clonal groups differing mainly in cell surface determinants (high degree of chromosomal recombination) and accessory genome components (plasmids and prophages, driven by horizontal gene transfer) (8, 9). In a separate adaptive trajectory from that of hypervirulent clones carrying low-level resistance, MDR K. pneumoniae are associated with the acquisition of plasmids, including large self-mobilizable types (e.g., FIIK), with complex multidrug resistance regions but relatively few virulence determinants (9–11). A correlation between plasmid type, carriage of antimicrobial resistance genes (e.g., *bla*_KPC_ or *bla*_CTX-M_) and specific clonal groups has been reported (11–13) but is not strict (14). It has been suggested that the observed genome plasticity of MDR clones may favor the acquisition of virulence factors (siderophore or hypermucoidy encoding genes, fimbrial loci etc.), leading to the emergence of resilient “hypervirulent MDR” clones (8, 9, 11). Tracking the epidemiology of MDR strains, particularly those belonging to clonal groups linked to disease outbreaks, is therefore critical.

Early accurate identification of the infective agent is crucial for timely and effective treatment of infections. While traditional typing methods, such as multilocus sequence typing or pulsed-field gel electrophoresis (PFGE), show low discriminatory power between distinct but closely related lineages, whole-genome sequencing (WGS) approaches have revolutionized outbreak detection, as well as antibiotic resistance surveillance (15, 16). However, WGS is not yet routinely used for investigation of single or limited infection episodes, especially in veterinary practice. Bacteriophages (phages) are viruses that specifically and selectively target bacteria and have long been used for typing pathogens (17) and as antimicrobial agents in therapeutic applications (18). Bacteriophage typing of bacteria can be a useful, rapid and cost-effective identification method, when WGS is not immediately accessible. Here, exploration of phage therapy options for a mare with severe respiratory disease, refractory to standard antibiotic treatment, led to the identification of varying bacteriophage susceptibility. Combined with antibiotic resistance profiling, this revealed multiple K. pneumoniae infecting strains in equine bronchial aspirates. Subsequent WGS identified five different coexisting MDR clones, four of which are recognized as globally disseminated emerging human pathogens.

## RESULTS

### Bacteriophage and antibiotic susceptibility profiles revealed a mixed K. pneumoniae population.

**FIG 1:**
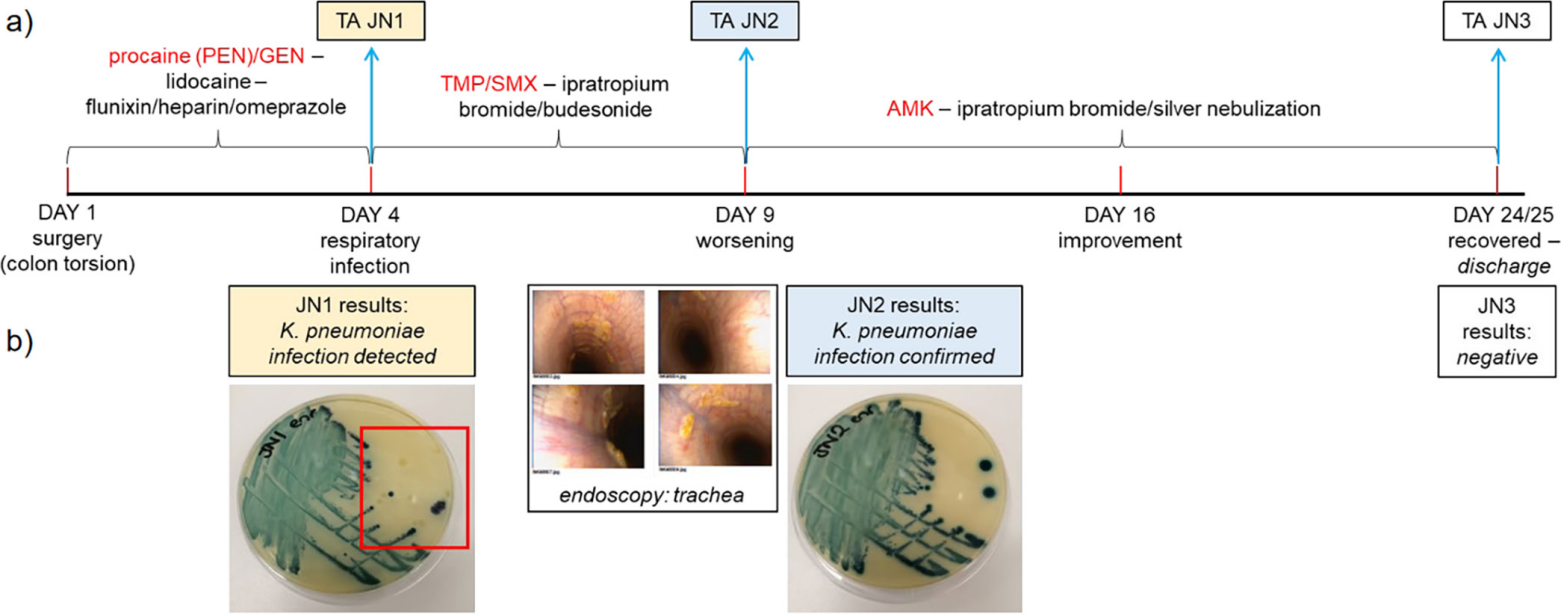
Infection time course for a horse with pneumonia. (a) Severe respiratory infection developed post-surgery in a 12 -year-old mare. Antibiotic treatment was prescribed immediately and optimized over the course of 4 weeks until the infection resolved after administration of costly amikacin (AMK). Peri- and post-operative care included intravenous fluids and lidocaine by constant rate infusion, regular gastric decompressions by nasogastric intubation, penicillin (intramuscular procaine (PEN), 22 mg/kg q12H), gentamicin (intravenous GEN, 6.6 mg/kg q24H), non-steroidal anti-inflammatory drugs (intravenous Flunixin, 1.1 mg/kg q12H), enoxaparin (subcutaneous Clexane 0,5mg/kg q24H), biosponge (Di-Tri-Octahedral Smectite, q8H orally), omeprazole (gastrogard, 4 mg/kg q24H oral) and altrenogest 0.22% (Regu-Mate, 11 mL q24H oral) for the 3 days. On day 4 post-surgery, a tracheal aspirate (TA JN1) was obtained and cultured on sheep’s blood agar. PEN/GEN was substituted by oral trimethoprim-sulfamethoxazole (TMP/SMX, 30 mg/kg q12H) and ipratropium bromide and budesonide nebulization. On day 9, endoscopy was performed (b) showing increased secretions in the trachea and results of JN1 testing confirmed K. pneumoniae infection. TMP/SMX treatment was stopped, silver nebulization started, in addition to continued ipratropium bromide and budesonide therapy, and a second TA specimen (JN2) was collected. On the following day, nebulized AMK treatment was started (3.3 mg/kg q24H). Although JN2 microbiology results confirmed K. pneumoniae infection (b), the horse’s condition steadily improved until discharge on day 25, when a third TA was collected (JN3; culture-negative). Microbiological testing on CHROMagar Orientation (b) confirmed the presence of K. pneumoniae among three other species (one colony each, red square) in JN1 and alone in JN2.

Culturing of tracheal aspirate (TA) JN1 (day 4, after PEN/GEN treatment) on chromogenic media revealed different colony morphologies with K. pneumoniae being overwhelmingly predominant. Three other sporadic (one colony each) morphotypes were also present, identified as Aeromonas caviae, Acinetobacter baumannii (neither previously reported in severe equine respiratory infections [19–22]) and E. coli (Fig. 1b). In TA JN2 (TMP/SMX treatment), collected 5 days later, K. pneumoniae was the sole species detected on standard CHROMagar. Nine bacteriophages were *de novo* isolated against seven randomly picked K. pneumoniae colonies (JN1 a-c and JN2 a-d) from environmental sources (*n* = 8; waterways, wastewater and feces) or TAs (*n* = 1, JIPh_Kp198) (Table 1). Six (JIPh_Kp 194–199) showed similar lytic activity against the seven hosts, while JIPh_Kp 192, 193, and 202 each lysed a different combination of isolates. JIPh_Kp 195, 192, 193 and 202 were therefore used to screen an additional 122 K. pneumoniae colonies (JN1 *n* = 61, and JN2 *n* = 61) (Table 1). Analysis of extended phage susceptibility profiles showed that the infecting K. pneumoniae population comprised at least three different predominant types and that the proportions of each changed over time. Profile C was predominant in JN1 (46%), declining in JN2 (3%), profile B remained relatively stable (34% in JN1 versus 55.5% in JN2), and profile G was detected in JN2 only (29%). A K. pneumoniae subset not susceptible to any of the bacteriophages tested was also identified in both specimens (profile D, 14% in JN1 and 11% in JN2) (Table 1). Three additional profiles, E (JN1), F (JN1) and H (JN2), were each associated with one isolate only (1.5%), while two isolates in JN1 (3%) were profile A (Table 1). Human clinical isolates SYD139 and SYD325 were both lysed by JIPh_Kp192 only (profile C).

**TABLE 1:** Bacteriophage susceptibility typing of K. pneumoniae isolated from the tracheal aspirates of a horse with pneumonia^*a*^

Profile	K. pneumoniae colony^*b*^	Phage lytic activity^*c*^					
		Profile	JIPh_ Kp192	JIPh_Kp193	JIPh_ Kp202	JIPh_Kp195	JIPh_Kp194, 196, 197, 198, 199
JN-1	**a** (ST307), **c** (ST307)	A	**Good**	Poor	None	None	None (**a, c**)
JN-1	**b** (ST628), 1, 2, 4, 5, 6, 9, 13, 21-23, 25, 33, 35-39, 49, 53-55	B	None	**Good**	None	**Good**	**Good** (**b**)
JN-1	d, 3, 7, 11, 12, 14-18, 24, 26, 29, 31, 32, 34, 40, **42** (ST307), 44, 46-48, 50, 52, 56-61	C	**Good**	None	None	None	*Nt*
JN-1	**8**, **10**, **19**, **20**, **27**, **30**, **41**, **45**, **51**	D	None	None	None	None	*Nt*
JN-1	**28**	E	None	Poor	None	Poor	*Nt*
JN-1	**43**	F	Poor	None	None	None	*Nt*
JN-2	**a** (ST628), **c** (ST628), 1, 3-5, 7, 9, 14, 18, 19, 22-24, 27-29, 31, 33-41, 44, 45, 49-51, 53, 54, 58, 59	B	None	**Good**	None	**Good**	**Good** (**a, c**)
JN-2	11, 12	C	**Good**	None	None	None	*Nt*
JN-2	**8**, **15** (ST392), **21**, **26** (ST4656), **42**, **52**, **60**	D	None	None	None	None	*Nt*
JN-2	**b** (ST893), **d** (ST893), 6, 10, 13, 16, 17, 20, 25, 30, 32, 43, 46-48, 55-57, 61	G	None	None	**Good**	None	None (**b, d**)
JN-2	**2**	H	None	None	Poor	None	*Nt*

a JIPh phages against K. pneumoniae were isolated *de novo* from sewage, human and animal feces, river water, and horse tracheal aspirates (TA).^*b*^In bold colonies with unique phage susceptibility profiles selected for further characterization including JN1 a, b, c and JN2 a, b, c, d (the seven strains initially used as targets for *de novo* bacteriophage isolation).^*c*^Good, complete lysis (clear spots); Poor, partial lysis (turbid spots); None, no lysis; *Nt*, not tested.

Antibiotic susceptibility testing of isolates (*n* = 27) with unique bacteriophage susceptibility profiles or resistant to all isolated bacteriophages confirmed differences among colonies (Table 2). All were resistant to cefotaxime (minimum inhibitory concentration (MIC) >4 μg/mL) and TMP/SMX (MIC >4 μg/mL), but susceptible to carbapenems (ertapenem MIC 0.06 μg/mL; meropenem MIC <0.06 μg/mL) and AMK (Table 2). Twelve isolates with unique combined (phage susceptibility/antibiotic resistance) profiles were further typed by PFGE (Fig. S1). 10 isolates with unique antibiotic/phage susceptibility and PFGE pattern were selected for WGS.

**TABLE 2:** Antibiotic susceptibility of selected equine K. pneumoniae isolates^*a*^

JN no.^*b*^ (phage susceptibility profile)	AMK	KAN	GEN	CHL	TET	CIP	FOX
**1_a (A)**	4	32	64	8–16	128–256	32	2–4
**1_b (B)**	2	32	32	8–16	128	0.5–2	2–4
**1_c (A)**	S	I	R	R	R	R	S
**1_42 (C)**	4	64	64	8–16	128	>2	4
**1_8 (D)**	4	32	>8	8–16	>64	>32	4
1_10 (D)	4	32	>8	8–16	>64	>32	4
1_19 (D)	4	32	>8	8–16	>64	>32	4–8
1_20 (D)	4	32	>8	8–16	>64	>32	4
1_27 (D)	4	32	>8	8–16	>64	>32	4–8
**1_28 (E)**	2–4	32–64	>8	16	>64	>32	4
1_30 (D)	4	32	>8	16	>64	>0.25	4
1_41 (D)	4	32	>8	16	>64	>32	4
**1_43 (F)**	4	32–64	>8	16	>64	>32	4
1_45 (D)	4	32	64	8–16	128	>2	4
1_51 (D)	4	32	>8	16	>64	>32	2–4
**2_a (B)**	2	16	32	8–16	128–256	0.5	4
**2_b (G)**	4	512–2048	64–128	256	128	2	4
**2_c (B)**	S	R	R	R	R	I	S
**2_d (G)**	S	R	R	R	R	R	S
**2_2 (H)**	2–4	>512	>8	256	>64	>32	2–4
2_8 (D)	2	32	32	8–16	256	0.5	4
**2_15 (D)**	4	64	128	8	256	>2	4–8
2_21 (D)	2	16–32	32–64	8–16	128	0.5	4
**2_26 (D)**	4	32	64–128	8	128	2	4
2_42 (D)	2–4	>512	>8	256	>64	>16	2–4
2_52 (D)	2	16	32	8–16	128–256	0.5	4
**2_60 (D)**	2	64	32–64	8–16	128	0.5	4

a Resistance phenotypes (R, resistant; I, intermediate; *S*, susceptible) and/or minimum inhibitory concentration (MIC, μg/mL) determined using EUCAST epidemiological cutoffs (66) for AMK, amikacin *S* ≤ 8, *R* > 16; GEN, gentamicin *S* ≤ 2, *R* > 4; CHL, chloramphenicol *S* ≤ 8, *R* ≥ 16; CIP, ciprofloxacin *S*  ≤ 0.25, *R* > 0.5; FOX, cefoxitin *S* ≤ 8, *R* > 8, or CLSI breakpoints (67) for KAN, kanamycin *S* ≤ 16, *R* ≥ 64; TET, tetracycline *S* ≤ 4, *R* ≥ 16. Shading: dark gray, resistant; light gray, intermediate; white, susceptible.^*b*^Bold, isolates chosen for further characterization.

### K. pneumoniae isolates belong to sequence types that cause disease in humans.

**FIG 2:**
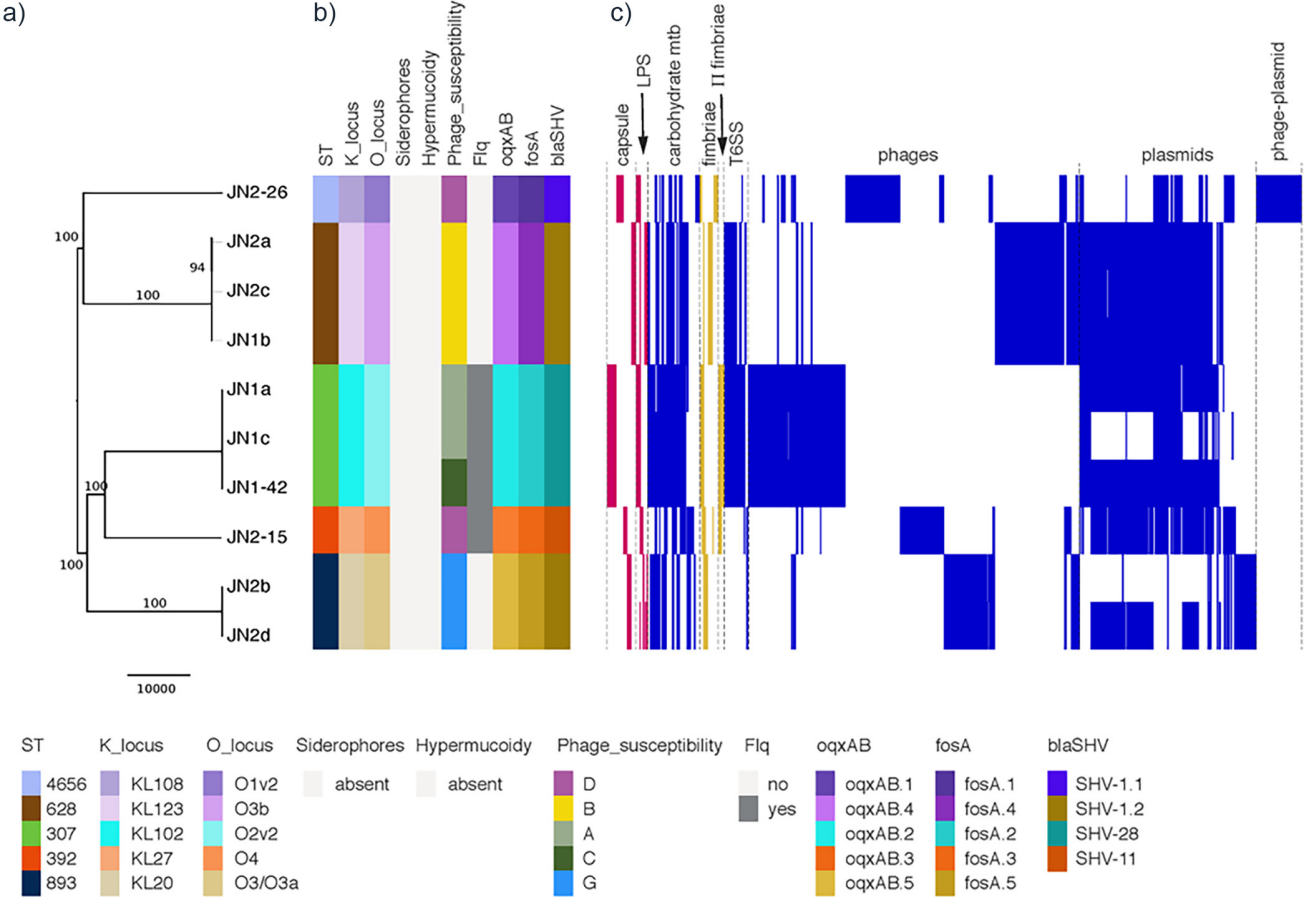
Phylogeny and comparison of accessory genome of K. pneumoniae isolates from equine tracheal aspirates. (a) Maximum likelihood phylogenetic tree (59,668 total single nucleotide polymorphisms (SNP) outside recombinant regions). Bootstrap values above 80 are shown on branches. Scale bar represents number of SNP. (b) Kleborate 0.2.0 (52) was used to type capsule, lipopolysaccharide (LPS), siderophores (yersiniabactin; colibactin; aerobactin; salmochelin) and hypermucoidy (*rmpA* gene). Chromosomal antibiotic resistance determinants intrinsic to K. pneumoniae: *fosA*, fosfomycin resistance; *oqxAB*, multidrug efflux pump; *bla*_SHV_, β-lactam resistance. Flq, chromosomal mutations (ParC-80I; GyrA-83I) associated with fluoroquinolone resistance. The corresponding color-coding chart is shown below the figure. (c) Selected regions of difference identified using Roary, reordered according to their synteny where possible. Regions corresponding to the accessory genome that could be confidently classified into the following functional categories (*n* = 1897 genes) are represented as follows: capsule and LPS (red); carbohydrate metabolism (blue); fimbriae and π-fimbriae (yellow); T6SS, Type VI secretion system; prophage-related regions; plasmid-related regions; and phage-plasmid related regions (blue). For clarity, regions of ambiguous function are not shown. Details of all regions are listed in Table S2.

The 10 sequenced isolates (*n* = 4 from JN1 [day 4, under PEN/GEN therapy]; *n* = 6 from JN2 [day 9, under TMP/SMX]) were grouped into five distinct sequence types (STs) by *in silico* MLST: ST 307 (JN1a, JN1c and JN1-42), 628 (JN1b, JN2a and JN2c), 893 (JN2b and JN2d), 392 (JN2-15) and the yet uncharacterized ST 4656 (JN2-26) (Fig. 2). ST 307, 628, 893 and 392 have all been described as emerging MDR clonal groups of concern for human health. ST307 isolates in JN1 presented multiple phage susceptibility profiles (A/C/D/F), while ST628 strains had profiles B/E (Table 1). In JN2, the only observed ST307-associated phage susceptibility profile was C (lowest frequency, 3%), while ST893 isolates, resistant to kanamycin, were first detected (profiles G/H). Profiles B and D, linked to ST628 by PFGE and WGS, were the most frequent in JN2 (66%).

Comparative genome analysis and screening for core virulence determinants confirmed the presence of intact capsular and lipopolysaccharide (LPS) loci specific to each ST, and of conserved virulence regions coding for enterobactin, acridine efflux pumps, Type 1 and Type 3 fimbriae, and a Type VI secretion system (T6SS locus I and III with minor variations) and its regulators (e.g., RscAB) (Fig. 2; Table S1; Table S2). None of the isolates carried the pullulanase-encoding operon (23), additional siderophores or hypervirulence-related genes (Table S2). However, as may be expected, there was significant variation between different STs in genes encoding outer membrane proteins and regions associated with carbohydrate metabolism and transport (Table S1). The T6SS locus II was present only in ST307, ST628 and ST4656, and the recently described K. pneumoniae π-fimbriae locus (11) was identified in both ST307 and ST392 (Table S1).

Comprehensive analysis of the pangenome using Roary showed that isolates of different STs varied in discrete regions correlating mainly with prophage and plasmid content, which were also the main differentiating elements between isolates of the same ST (Table S1; Fig. 2). ST893, ST392 and ST4656 isolates carry a Type I-E CRISPR-Cas locus (Table S1) with small differences in the *cas* genes and different spacer arrays. ST307 isolates all carry an additional capsular locus (Cp2), previously described in human ST307 isolates (11, 24) (Table S1; Table S3). These genomic differences within and between STs are expected to result in the observed variation in susceptibly to exogenous bacteriophage infection.

### The genomes of K. pneumoniae ST307 from horse and humans do not differ substantially.

**FIG 3:**
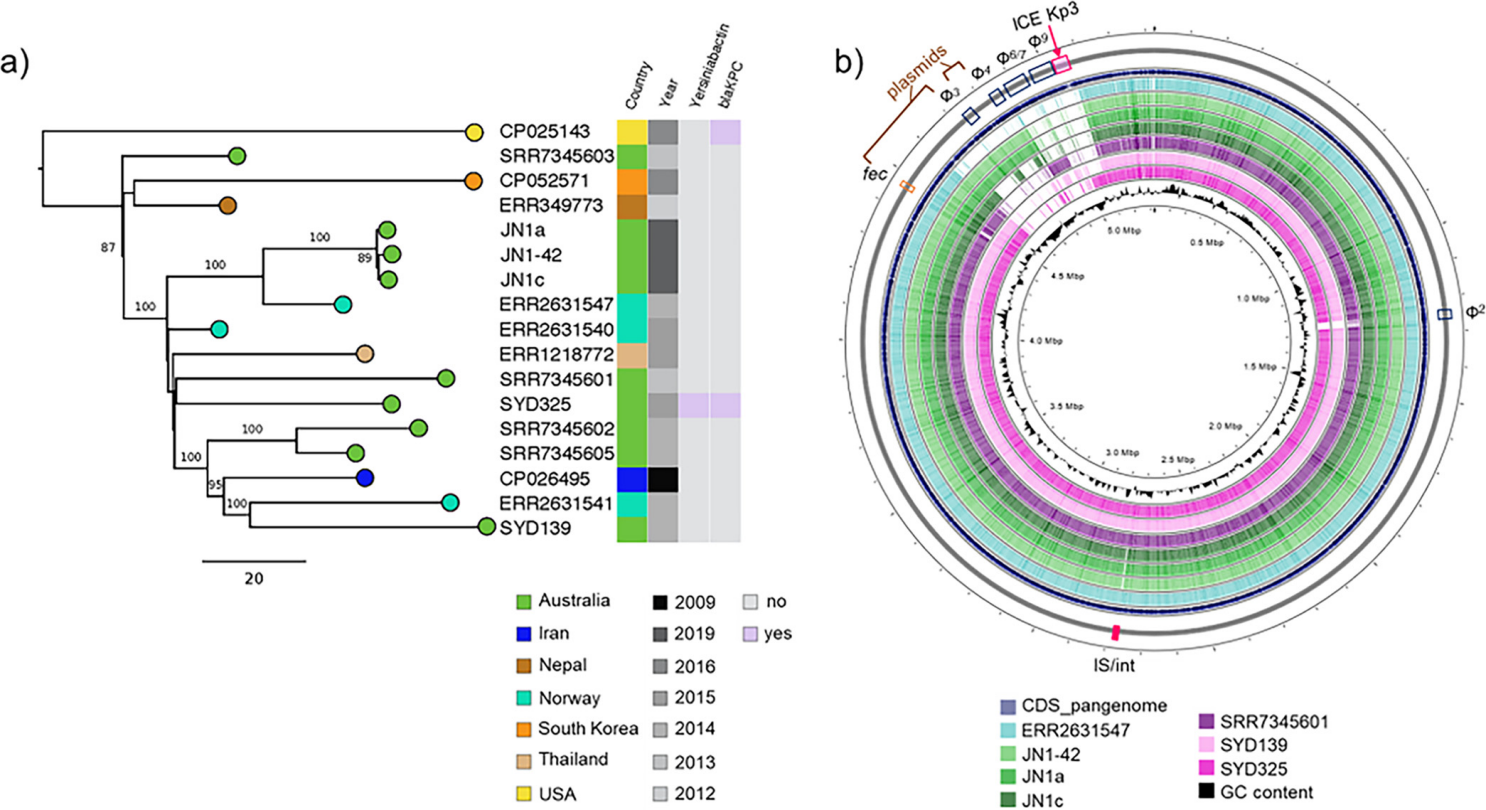
Phylogeny of K. pneumoniae ST307 equine and human isolates. (a) Maximum likelihood phylogenetic tree (550 total single nucleotide polymorphisms (SNP) outside recombinant regions). Bootstrap values above 80 are shown on branches. Scale bar represents number of SNP. The genomes of three ST307 equine isolates were compared to those of 14 K. pneumoniae ST307 isolated from humans worldwide. SNP analysis was conducted by whole-genome alignment against K. pneumoniae Kp616 (GenBank CP026495; 24) using Snippy v3.1 (https://github.com/tseemann/snippy). (b) Comparative genomic analysis of local ST307 isolates from horse (JN1-42, 1a and 1c) and humans (SYD139 and 325), and closely related human isolates (GenBank ERR2631547 and SRR7345601). The map was generated in CGView (65) by alignment of the assembled genomes against the complete set of features in the ST307 pangenome (reference.fa from Panaroo; 56). The analysis identified few differences, mainly associated with prophage (Φ) and plasmid content, as well as the presence of an integrative chromosomal element (ICEKp3) carrying genes encoding the yersiniabactin siderophore in SYD325. IS/int, insertion sequences and integrases. Fec, ferric citrate transport operon. Details of all regions are listed in Table S3. Color-coding charts are shown below each panel.

The availability of WGS data for ST307 K. pneumoniae in public databases enabled comparison of equine ST307 genomes with 12 representatives of the global human ST307 population and with two human ST307 strains (SYD139 and SYD325) from our own clinical collection. Our isolates clustered with a Norwegian isolate (ERR2631547) from human blood (24) but were separate from other sequenced human isolates from Australia (Fig. 3a). The main genomic regions of difference between ST307 isolates identified using Panaroo were again linked to plasmid and prophage content (Fig. 3b; Table S3). There was no appreciable difference between equine and closely related human isolates in terms of antibiotic resistance or virulence gene carriage, except for the presence in SYD325 of the yersiniabactin encoding locus and of *bla*_KPC-3_, encoding carbapenem resistance (Fig. 3b).

### Multidrug resistance of K. pneumoniae isolates correlates with identified resistance genes.

**FIG 4:**
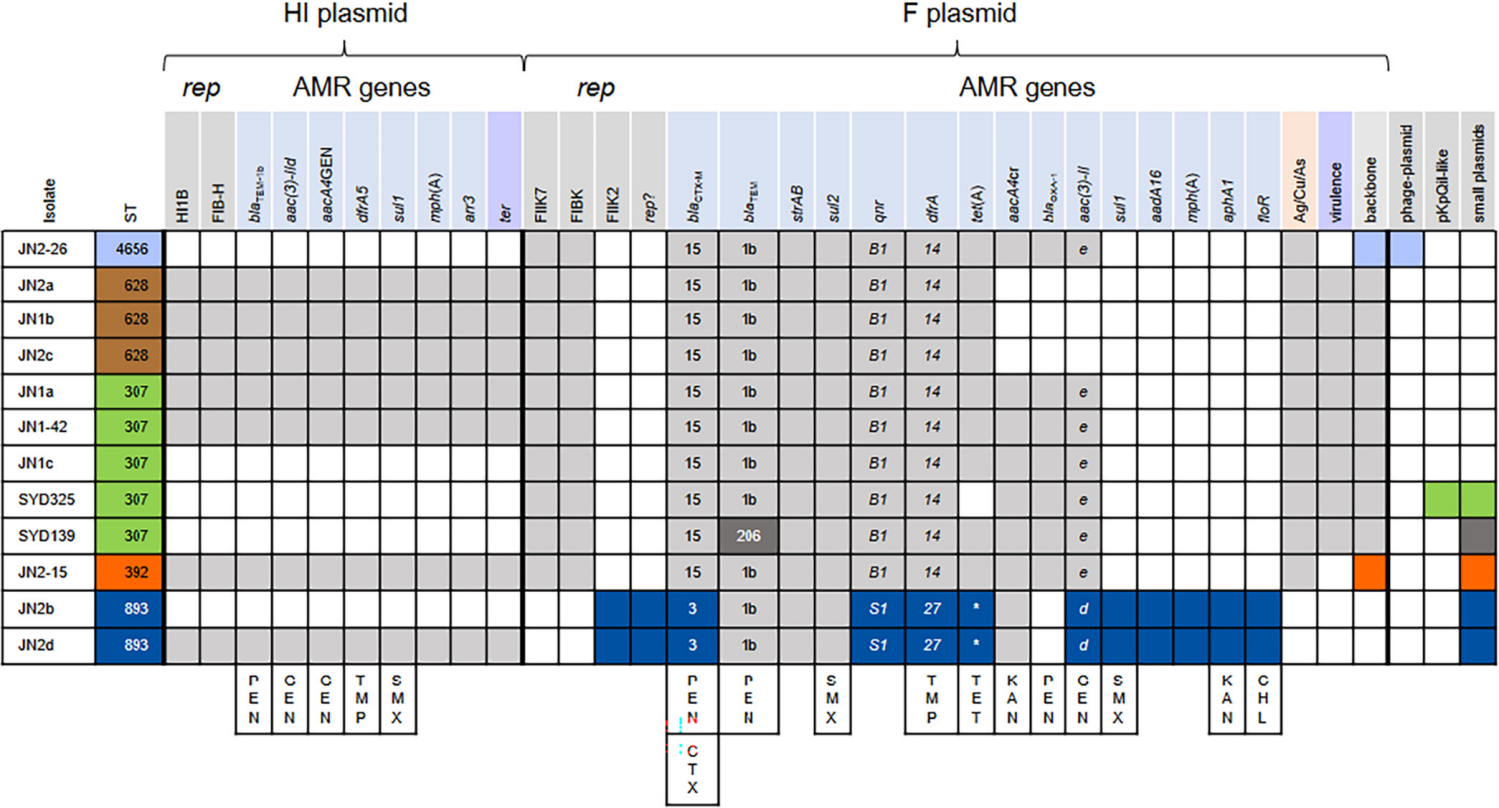
Correlation of antibiotic resistance gene content, plasmid replicons and predicted resistance phenotypes. *rep*, all from PlasmidFinder (59); HIB 567/570 nucleotides identical to “IncHI1B(pNDM-MAR)”; FIB-H, identical to “IncFIB(pNDM-MAR)”; FIIK7, FIIK-type replicon, K7 by RST (59); FIB(K), FIB(K)-type replicon; FIIK2, FII(K)-type replicon, K2 by RST; *rep?*, replication initiation gene of unnamed type. Antibiotic resistance (AMR) genes are organized according to proposed plasmid location (as shown in Fig. S2 and S3). Gene variants identified by mapping raw reads are specified. *, in the *tet*(A) column indicates that the last 9 amino acids of the Tet(A) protein are different due to truncation of the *tet*(A) gene. *ter*, region associated with resistance to stress induced by resident gut microflora (28); Ag/Cu/As, silver, copper and arsenic resistance regions; virulence, urea ABC-transport, Fec-like iron (III) dicitrate transport, glutathione ABC-transporter; *lacIZY*, glycogen synthesis; backbone, backbone type (see Fig. S2); phage-plasmid, phage element with a region 95% identical to PlasmdFinder “IncFIB(pKPHS1)” target; pKpQIL, pKpQIL-like plasmid carrying *bla*_KPC-3_. Last column, small plasmids were found as circular contigs: SYD325, 34.032 kb N3-type plasmid plus 1.546 kb plasmid; SYD139, 3.991 kb plasmid (~40 copies/chromosome) matching one found in K. pneumoniae, and 3.223 kb plasmid (~37 copies/chromosome), closely related to plasmids found in various Enterobacterales; JN2-15, 1.552 kb plasmid (~50 copies/chromosome) identical to plasmids found mainly in E. coli; JN2b and JN2d, 5.617 kb plasmid with no close matches and 5.251 kb plasmid almost identical to plasmids from K. pneumoniae and other species (both ~60 copies/chromosome). Relevant resistance phenotypes conferred by selected resistance genes are shown at the bottom: PEN, penicillin; GEN, gentamicin; TMP, trimethoprim; SMX, sulfamethoxazole; CTX, cefotaxime; TET, tetracycline; KAN, kanamycin; CHL, chloramphenicol. *bla*_CTX-M-15_ would be expected to confer resistance to ceftazidime in addition to CTX, while *bla*_CTX-M-3_ would not. *qnrB1*, *qnrS1* and *aacA4*cr could all contribute to fluoroquinolone resistance.

As expected, *fosA* (fosfomycin resistance), *oqxAB* (multidrug efflux pump) and *bla*_SHV_ (β-lactam resistance) genes intrinsic to K. pneumoniae were found on the chromosome and variants correlate with ST (Fig. 2). ST307 and ST392 isolates have chromosomal mutations (ParC-80I; GyrA-83I) linked to fluoroquinolone resistance (Fig. 2a). All other antibiotic resistance genes appear to be carried on plasmids (Fig. 4). Each isolate carries a set of resistance genes expected to give resistance to all antibiotics used unsuccessfully to treat the horse, but not to AMK (Fig. 2a and 4). In all isolates, resistance to PEN could be conferred by *bla*_TEM,_ *bla*_CTX-M_ and/or the chromosomal *bla*_SHV_ gene, GEN by *aac(3)-II* genes, with *aacA4*GEN (a variant of *aac(6′)-Ib* with C at position 329 of the gene cassette) also potentially contributing, and TMP/SMX by a *dfrA* gene plus a *sul* gene. JN2b (ST893) had the highest kanamycin and chloramphenicol MICs, likely due to *aphA1* and *floR*, respectively. Fluoroquinolone resistance seen in some isolates is likely due to a *qnr* gene (low-level-resistance), found in all isolates, and/or an *aac(6′)-Ib*-cr variant (called *aacA4*cr here; low-level resistance), found in most isolates, and ParC-80I and GyrA-83I chromosomal mutations.

### K. pneumoniae isolates carry closely related plasmids.

An FIIK7-type and an FIBK-type replicon, related to pKPN3-307_type A plasmids (11, 24), were identified in sequenced isolates of all ST except ST893. Putative plasmids (pJN#_F) containing both replicons could be assembled for all except SYD325, which also has a pKpQIL-like plasmid with partially similar sequence. All pJN#_F plasmids carry arsenic and copper/silver resistance clusters found in pKPN3-307_type A. Plasmids in ST307 and ST628 also have five regions proposed to contribute to virulence (glycogen synthesis, *lacYZI*, glutathione ABC-transport, Fec-like iron (III) dicitrate transport and urea ABC-transport) (11) downstream of the *rep*FIIK gene (Fig. S2).

pJN2-15_F (ST392) and JN2-26_F (ST4656) lack these virulence-associated regions and have regions of dissimilarity compared with the pJN#-F plasmids in ST307/ST628 and with each other (Fig. S2). JN2-26_F (~190 kb) is closely related to pE16K0288-1 (K. pneumoniae ST45, GenBank CP052263; 25) and pJN2-15_F (ST392) to pE16KP0133-1 (K. pneumoniae ST392 from human blood; 25). pJN_F plasmids from ST628 isolates lack an IS*26*-flanked region carrying resistance genes compared with those from other STs (Fig. S2). These differences suggest that the related FIIK7-FIBK plasmids were already present in each ST, rather than a single plasmid being transferred between different STs during the infection.

The FIIK2-type replicon identified in ST893 is found on ~109 kb plasmids (pJN2b_F and pJN2d_F) very closely related to pSCM96-1 (GenBank CP028717; K. pneumoniae ST15 isolated from human sputum; 26) (Fig. S3). A 26 kb deletion in the *tra* region of these plasmids versus pSCM96-1 suggests that pJN2b_F are pJN2d_F are unlikely to be conjugative. pJN2b_F and pJN2d_F have some resistance genes (*bla*_TEM_, *strAB*, *sul2*) in common with the FIIK7-FIBK plasmids in the other STs. Other genes are different but confer resistance to the same antibiotics (e.g., *drfA14* versus *drfA27* trimethoprim resistance, and *aac(*3*)-IIe* and *aac(*3*)-IId* gentamicin resistance) (Fig. 4).

PlasmidFinder IncFIB(pNDM-MAR) and IncHI1B(pNDM-MAR)-like targets were also identified in seven isolates, of all STs except ST4656 (JN2-26). The same proposed ~230 kb plasmid (pJN_HI; Fig. S4) was assembled for all isolates except JN1a, where S1/PFGE suggests a larger HI plasmid of similar size to that of the ST307-type FIIK plasmid. pJN_HI would be classified as HI3 (27) and, while the organization of the backbone is related to available plasmid sequences, there are no close matches over the whole plasmid. pJN-HI carries antibiotic resistance genes including *bla*_TEM_ and *drfA5*, and a *ter* operon (Fig. S4; Table S1), originally named for tellurite resistance but recently reported as being associated with infection and enhanced fitness during gut colonization (28).

Small, high copy number plasmids were also present in ST893, ST392 and ST307 human clinical isolates (Fig. 4). A 109.952 kb circular contig obtained for JN2-26 (named pJN_2-26) is a phage-like plasmid with high sequence identity to Klebsiella phage ST13-OXA48phi12.3 (GenBank MK422451) and Salmonella phage SSU5 (NC018843) (both family *Siphoviridae*), and similarity to several other K. pneumoniae mobile elements (e.g., CP016161, MF144193, and CP015755). The plasmid-related loci of pJN2-26 include a *rep* gene with 95% identity to the PlasmidFinder FIB(pKPHS1) target.

### K. pneumoniae isolates of different ST do not show significant differences in fitness or adhesion properties.

As indicators of relative fitness, we compared the growth rate in rich media (LB) and biofilm production for the sequenced equine K. pneumoniae isolates, and the ability of a representative of each predominant ST [ST307, JN1-42; ST628, JN1b; ST893, JN2b] plus human isolates SYD139 and SYD325 to adhere to T24 human bladder epithelial cell monolayers. We found no significant difference in growth rate, biofilm forming ability or epithelial adherence among the tested isolates (Fig. S5 a-c). Adhesion levels were comparable for equine and human strains (10^6^ CFU) and matched those previously reported for pathogenic K. pneumoniae (29).

## DISCUSSION

**FIG 5:**
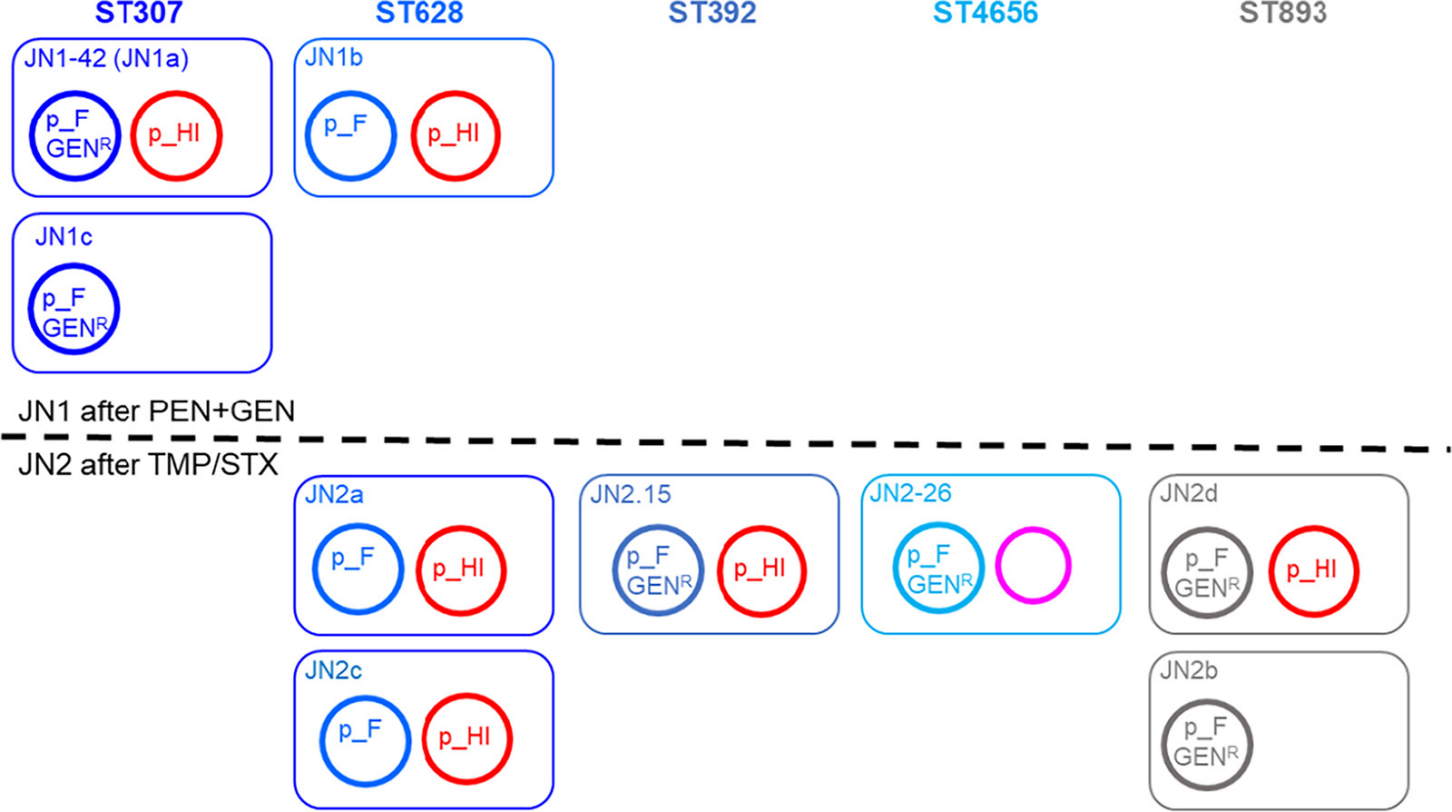
Summary of sequence types and plasmid content in JN1 and JN2 specimens. Rectangular boxes of different colors represent cells of different ST, as indicated. Related F plasmids, shown in different shades of blue, are labeled. Distinct F plasmids in ST893 are shown in gray. All are expected to confer resistance to penicillins (PEN) and to trimethoprim- sulfamethoxazole (TMP/SMX). F plasmids in all except ST628 are expected to confer gentamicin (GEN) resistance, as indicated. The HI plasmid, shown in red, is expected to confer PEN, GEN and TMP/SMX resistance (JN1a seems to have a slightly larger variant). JN2-26 only has a phage-plasmid (pink), which does not carry any resistance genes.

Post-operative bacterial pneumonia can be a serious complication in horses (21, 22), with general anesthesia and compression of the lungs being two important risk factors (19, 30). The etiology of equine pleuripneumonia is mostly linked with the translocation of common oropharyngeal flora (e.g., Streptococcus) from the upper airways and mouth into the lower respiratory tract (19), but enteric Gram-negative species such as K. pneumoniae can also be causative agents, most often introduced from the immediate external environment (6, 20–22). For this study, tracheal aspirates were obtained from a mare with severe post-surgery respiratory disease due to K. pneumoniae refractory to routine antibiotic therapy but responsive to AMK, which is costly and infrequently used in horses (31). The origin of the infecting MDR strains could not be definitively established as Klebsiella were not isolated from environmental samples tested for routine infection control at the veterinary clinic, and no feed or fecal samples, often Klebsiella reservoirs and likely alternative source, were analyzed. It is not uncommon, even in human settings (32), for the detection of the origin of the infecting Klebsiella to be rather problematic, as this is a ubiquitous, adaptable and widely distributed environmental species (4). Thus, rapid identification and characterization of antibiotic resistance phenotypes in the infecting strain becomes crucial in ensuring effective treatment and avoiding exacerbation of disease by inappropriate antibiotic choice. Antibiotic pressure is also one of the most important drivers of genetic recombination facilitating horizontal transfer of resistance determinants, which can further compromise therapeutic success (8, 33). Here, the antimicrobial therapy given may in fact have sustained K. pneumoniae population growth and diversification at the site of infection (i.e., more STs detected over time (JN1 versus JN2) carrying more diverse plasmids; Fig. 5), where coexisting strains could exchange or acquire advantageous genetic traits.

Initially, bacteriophage therapy was considered as alternative to AMK administration, and we were able to isolate several lytic bacteriophages against K. pneumoniae from bronchial specimens. Bacteriophage typing, combined with extensive sampling and detailed antibiotic susceptibility testing, revealed several different MDR K. pneumoniae coexisting at the site of infection, each potentially able to contribute to disease progression and persistence. Outbreaks involving multiple K. pneumoniae clones have been reported (15, 34, 35), but there is little evidence of multi-clonal infections in the same host other than this study. Despite knowledge of the occurrence of mixed microbial infections, it is too often readily accepted for one dominant virulent clone to outcompete “weaker” members (STs) of the same species in each infection event. However, in this study, using deep sampling techniques, we identified different STs with the same potential for virulence and similar resistance profiles coexisting during infection, reinforcing the observation that standard sampling protocols may be inadequate in capturing the complexity of microbial infection niches (36).

The genomic features of ST4656 are first described here, while the remaining four clones (ST307, ST628, ST392 and ST893) belong to pathogenic groups considered emerging human health threats and recently reported separately as causes of severe infection (including sepsis and pneumonia; 24, 37–40) or disease outbreaks (34, 35, 41, 42) in humans worldwide. ST307 and ST893 have also been reported in other animal infections (e.g., urinary tract infections), particularly in companion animals (43, 44). Comparative analyses of ST307 isolates from humans has previously shown variability in accessory genome components within this clone, while it has been proposed that the acquisition of specific genetic traits, such as π fimbriae, may have contributed to its successful adaptation to specific human niches, such as the urinary tract, or to nutrient-limiting conditions (11, 24, 39, 40). The equine ST307 isolates from our study have all the characteristics of clade V human ST307 carrying *bla*_CTX-M-15_ as defined by Lowe et al. (40), based on chromosomal mutations associated with fluoroquinolone resistance and prophage component.

This heterogeneity is reflected in our isolates, with some virulence factors described in human ST307 (11, 24) found in both equine isolates and local clinical strains SYD139 and SYD325 (π fimbriae and Cp2), others only in SYD325 (yersiniabactin/Type IV secretion locus), and others (e.g., hypervirulence loci) completely absent. Except for an HI-type plasmid not yet reported in humans, our study showed that horse and human ST307 isolates are closely related, and that genomic differences between them are independent of source species. We also showed that equine and local human ST307 isolates, as well as ST628 and ST893, have the same ability to adhere to epithelial cells, at levels equivalent to those previously reported for a clinical highly pathogenic K. pneumoniae from a human patient with a urinary tract infection (29).

To our knowledge, this is the first study to directly compare genomic features of different STs isolated from the same infection site. We showed that all characterized K. pneumoniae have similar virulence attributes and may have equivalent capacity to cause infection. However, subtle differences in metabolism or antibiotic resistance profiles may allow one pathogen to replace the other as environmental conditions and treatments change, prolonging or worsening disease symptoms. The differentiating features associated with pathogenicity that may play a role under changing conditions during cooperative infection could now be further explored through functional *in vitro* and *in vivo* studies (45).

The presence of the MDR HI-type plasmid in all but one (ST4656) of these equine isolates is strong evidence of horizontal transfer likely during the protracted coexistence of these different clones in the same niche. In humans the successful dissemination of MDR K. pneumoniae clonal groups has been correlated primarily with plasmid acquisition (8, 11, 25, 33). In this study, the main differences among STs were mainly in the plasmid components. ST307 and ST628 isolates carry related MDR F-type plasmids with the same accessory genes likely to favor niche specialization (silver, copper and arsenic resistance; urea and iron transport, glycogen metabolism etc.). Some of these key operons associated with virulence are missing from ST893, ST392 and ST4656 F-type plasmids, but in all STs multidrug resistance is associated with plasmid carriage. We showed that some horse MDR F-type plasmids are almost identical to those found in isolates from humans (11, 24) and that others are clearly related (25, 26). Not surprisingly, multidrug resistance regions similar to those seen in the equine plasmids have also been described before in plasmids from human isolates.

K. pneumoniae resistant to carbapenems or encoding extended spectrum β-lactamases (ESBL) (2, 3, 8, 11, 24, 33, 35) are cause of serious outbreaks of human disease and are rapidly becoming more common in horse infections (42, 43); in fact, all equine isolates in this study carry a plasmid-associated *bla*_CTX-M_ ESBL gene. Our findings support the fundamental role in K. pneumoniae adaptation attributed to plasmids (10, 15, 41) and evidence that the pool of resistance and virulence genes is diverse and widely accessible to different subtypes. Although our sample size was relatively small (*n* = 10 isolates) and a more detailed comparative analysis could be performed on larger populations, our findings demonstrate the close relationship between animal and human K. pneumoniae reservoirs, with important implications for epidemiology and associated infection control policies.

This study highlights the value of careful screening strategies and smart antibiotic choices for successful therapy, suggesting that testing of multiple colonies from diagnostic specimens should be more routine and that re-introduction of phage typing as an additional screening method, particularly in settings where WGS costs cannot be met, could be beneficial.

## MATERIALS AND METHODS

### Clinical case.

A pregnant 12-year-old mare (600 kg) was admitted for acute and severe colic to the Camden Equine Centre, Sydney School of Veterinary Science, University of Sydney (NSW, Australia), where a colon torsion was treated by exploratory laparotomy. Peri- and post-operative care was as per standard protocols (Fig. 1), including antibiotic therapy with intramuscular penicillin (procaine, PEN) and intravenous gentamicin (GEN) for the first 3 days. On day 4 post-surgery, the mare developed a respiratory infection, characterized by fever, tachypnoea and mild respiratory distress, so PEN/GEN therapy was replaced with oral trimethoprim-sulfamethoxazole (TMP/SMX) and nebulization with ipratropium bromide and budesonide (Fig. 1a). At this time, a tracheal aspirate (TA; JN1) was sampled for bacterial growth on sheep’s blood agar (SBA) with overnight culture. Proteomic fingerprinting (MALDI Biotyper System, Bruker, Preston, Victoria) confirmed K. pneumoniae as the primary infectious agent (Fig. 1b). After a brief initial improvement, the mare’s tachypnoea worsened. TMP/SMX treatment was stopped, and silver nebulization started in addition to continued ipratropium bromide and budesonide therapy, and a second TA specimen (JN2, day 9) was collected. On day 10, last-line amikacin treatment was started (nebulized AMK, 3.3 mg/kg every 24 h). Microbiological analysis of JN2 confirmed continuing infection with K. pneumoniae (Fig. 1b), but AMK therapy resulted in slow improvement of disease symptoms over 2 weeks, and a third TA (JN3) on day 24 confirmed resolution of the infection (no bacterial growth on SBA). AMK was discontinued and the mare was discharged from the veterinary hospital on day 25. A low grade, asthma-like residual respiratory inflammation was observed at discharge, but this was successfully treated to full recovery with corticosteroids (oral prednisolone and clenbuterol) and bronchodilators at home. The course of infection, treatment details and outcomes are outlined in Fig. 1.

### Isolation of bacteria and bacteriophages from equine tracheal aspirates.

Bacterial isolation and presumptive identification were performed at The Westmead Institute for Medical Research by direct plating of TAs JN1 and JN2 on chromogenic agar (CHROMagar Orientation, CHROMagar, Paris, France) (Figure 1b). Bacterial colonies of different morphology and color were typed using the MALDI Biotyper System following manufacturer’s instructions (Bruker Pty. LTD., Preston, Victoria). Three K. pneumoniae from JN1 (a-c) and four from JN2 (a-d) were used as targets for *de novo* bacteriophage isolation from equine TAs and from specimens previously obtained from various environmental sources (e.g., feces, waterways, sewage etc.) using the enrichment technique as before (46). Briefly, filtrates obtained by centrifugation (4 700 g, 10 min; Thermo Scientific Heraeus Multifuge X3R, rotor 75003180) and filtration (0.22 μm membrane filters; Millipore, Burlington, MA, US) were incubated overnight (225 rpm, 37°C) with the target bacteria grown to logarithmic phase (OD_600_ 0.4–0.6) in lysogeny broth (LB; Becton and Dickinson, Sparks, MD, USA). Bacterial debris was pelleted by centrifugation and aliquots of bacteriophage-enriched filtrates were embedded in soft top agar (LB 0.7%) for double-layer plaque assays (47). Single plaques were purified by serial passage (3x) prior to preparation of high concentration filtrates in SM buffer (50 mM Tris-HCl, 8 mM MgSO_4_, 100 mM NaCl, pH 7.4) (47). Four *de novo* isolated bacteriophages were used to rapidly type (spot testing on LB agar double layers) an additional 122 K. pneumoniae colonies from both specimens [*n* = 61 from JN1; *n* = 61 from JN2] and two ST307 isolates from our clinical human collection, SYD139 and SYD325. Clinical MDR K. pneumoniae (*n* = 11) of various sequence types (ST), K. pneumoniae ATCC 13883, Escherichia coli ATCC 25922, and clinical isolates from our diagnostic collections (including K. oxytoca, extra-intestinal MDR E. coli ST131 and human gut commensal E. coli) were used as negative controls to confirm bacteriophage specificity.

### Characterization of K. pneumoniae isolates. (i) Microbiology and whole-genome sequencing.

A subset of isolates with unique bacteriophage susceptibility profiles or resistant to all phages tested (*n* = 19), plus JN1 a-c and JN2 a-d were selected for further characterization. Minimum inhibitory concentration (MIC) for a panel of antibiotics, comprising the main clinical classes, was determined using the microtiter plate broth dilution assay as before (48, 49). Twelve isolates with a unique combined antibiotic resistance/phage susceptibility profile were typed by PFGE as before (50). 10 isolates chosen on the basis of unique PFGE fingerprint, representing each phage/antibiotic resistance profile, and JN1 a-c and JN2 a-d, used for bacteriophage isolation, were sequenced by Illumina NextSeq (paired-end; 2 × 150 bp; NextSeq 500 NCS v2.0).

Bacterial DNA was extracted and purified using the DNeasy Blood and Tissue DNA isolation kit (Qiagen, Hilden, Germany). WGS was performed as previously described (46). Our analysis workflow based on publicly available tools was used for *de novo* assembly of sequencing reads and simulated reads of NCBI reference genomes (Unicycler v.0.4.8; 51) to determine sequence type (*in silico* MLST), virulence genotypes, and Klebsiella specific capsule and LPS locus types (Kleborate v 0.2.0; 52). Variant calling was performed using Snippy v3.1 (https://github.com/tseemann/snippy) and recombination identified using Gubbins (46). Phylogeny was determined using IQ-TREE v.1.6.7 (53) (substitution model: GTR+I+G; 1000 bootstrap replicates). Phylogenetic trees and metadata were visualized with Microreact (54; https://microreact.org). The genomes of two local ST307 clinical isolates, SYD139 and SYD325, from our own collections, and 12 publicly available genome sequences representative of the global ST307 K. pneumoniae population (GenBank accessions: CP026495, ERR1218772, ERR349773, ERR2631540, ERR2631541, ERR2631547, SRR7345601, SRR7345602, SRR7345603, SRR7345605, CP052571, CP025143) were used for comparative analysis with the three equine ST307 isolates (JN1a, JN1c and JN1-42). SNP analysis was conducted by whole-genome alignment against one of these, K. pneumoniae Kp616 (GenBank CP026495; 24). Roary v 3.11.0 (55) and Panaroo (56) were used to identify regions of difference across isolates, based on contiguity and functional categories of the genes present. Outputs were curated using BLAST (57), PHASTER (58) and SnapGene software (Insightful Science; available at snapgene.com) for manual checking and annotation.

### (ii) Plasmid analysis.

To examine plasmid content prior to WGS, the same 12 isolates used for PFGE (with unique combined antibiotic resistance/phage susceptibility profiles) were treated with S1 nuclease (Promega, Madison, WI, USA) followed by PFGE as before (46). Replicon types were identified in Illumina data using PlasmidFinder (59) and, for F-type replicons, further distinguished using replicon sequence typing (RST) (59; http://cge.cbs.dtu.dk/services/pMLST/). Antibiotic resistance genes and associated mobile elements were annotated using Gallileo AMR (GAMR, formerly MARA) (60). This was coupled with analysis of Unicycler assembly graphs (Bandage 0.8.1; 61) and reference mapping (Geneious R9.1.8; https://www.geneious.com) to infer plasmid sequences from Illumina data. This was possible because: i) some isolates contained a single plasmid, ii) suitable reference plasmid sequences were available for comparison, iii) different isolates carried closely related plasmids, and iv) where two plasmids were present, backbone components were quite different, except for SYD325. PCR was used to link across problematic backbone regions (repeated elements) that broke contigs.

IS*26* is present in multiple copies in all isolates, resulting in splitting of contigs carrying resistance genes at IS*26* boundaries. IS*26*-flanked segments were assigned to plasmids based on correlations with the presence/absence of particular replicons over the whole set of isolates. Segments flanked by IS*26* were ordered by comparison to reference plasmid sequences and/or available multidrug resistance regions. These segments can potentially move between plasmids, but proposed plasmid sizes agreed with S1/PFGE results. Minor differences between related plasmids and resistance gene variants were checked by alignment of raw reads to contigs and corrected as necessary. Plasmid genes were annotated with reference to available plasmid sequences (11, 25). The circular genome of the phage-plasmid element found in JN2-26 was annotated using RAST-tk (62), BLAST (57) and PHASTER (58).

### *In vitro* fitness and virulence assays. (i) Growth rate, biofilm production and adhesion.

Growth was assessed as before (49). Briefly, 1:1000 dilutions of overnight bacterial cultures (LB) were incubated at 37°C with gentle orbital shaking in 96-well microtiter plates (Corning Incorporated, Durham, NC, USA) and kinetics over 18 h were measured in a Vmax Kinetic microplate reader (Molecular Devices, San Diego, CA, USA) (OD_600_; every 5 min). Biofilm assays were performed as previously (46) with minor modifications: polystyrene tissue culture microtiter plates were used and K. pneumoniae was grown in tryptic soy broth (TSB) and incubated for >20 h at 37°C (63). Both experiments included eight technical replicates and were repeated at least twice.

Bacterial adhesion was estimated as previously (64). Briefly, T24 human bladder epithelial cells (ATCC HTB-4), routinely maintained in McCoy’s 5A media supplemented with 10% fetal bovine serum (FBS) (Invitrogen, Waltham, MA, USA), were seeded at 4.2 × 10^5^ cells/well in a 24-well plate and allowed to grow to confluence overnight. Bacterial strains were subcultured 1:100 in LB and grown to mid-exponential phase (OD_600_ 0.4–0.6). T24 cell monolayers were infected with bacteria at a multiplicity of infection of 30 for 1 h at 37°C with 5% CO_2_. Non-adherent bacteria were removed by washing in phosphate-buffered saline (PBS) three times. Cell monolayers were then lysed with 0.1% (vol/vol) Triton X-100 in PBS. The number of cell-associated bacteria was determined by spot plating serially diluted cell lysates. Each strain was assessed in duplicate wells and assays were repeated five times.

### Data availability.

All data sets are included in this article and its Supplementary Material. Illumina raw reads for all equine K. pneumoniae isolates and human isolates SYD139 and SYD325 were deposited in the SRA (NCBI) database (BioProject PRJNA751993; BioSamples: SAMN20566092 to SAMN20566103). The annotated sequence of pJN2-26 was deposited in GenBank under MZ779062.
