# Supplementary material for: Co-Occurrence of Multidrug Resistant Klebsiella pneumoniae Pathogenic Clones of Human Relevance in an Equine Pneumonia Case
Source: Microbiol Spectr. 2022 May 17;10(3):e02158-21. doi: 10.1128/spectrum.02158-21 (PMC9241755; doi:10.1128/spectrum.02158-21)
Supplement: SUPPLEMENTAL FILE 4 — Supplemental material. Download spectrum.02158-21-s004.pdf, PDF file, 1 MB [file spectrum.02158-21-s004.pdf]

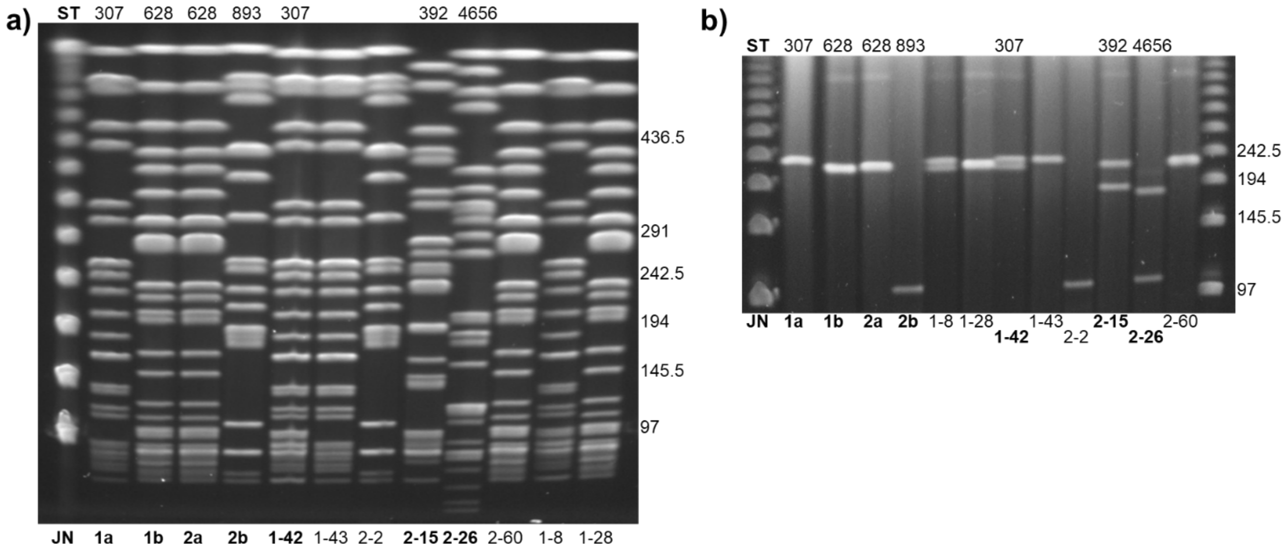

Figure S1. Genomic profiles of *K. pneumoniae* isolates. Pulsed field gel electrophoresis of (**a**) *XbaI* digested and (**b**) S1 nuclease treated (to visualise plasmids) genomic DNA of *K. pneumoniae* from equine tracheal aspirates. Isolates with unique profiles (in **bold**) were selected for whole genome sequencing. **(b)** Plasmid sizes predicted from sequence assembly are: HI plasmid (JN1b, JN2a, JN2c, JN1-42, JN2-15), ~230 kb; pJN1b_F/pJN2a_F/JN2c_F, ~238 kb (runs with HI plasmid, giving a single, brighter band); pJN2b_F, ~110 kb; pJN1-42_F, ~247 kb; pJN2-15_F, ~196 kb; pJN2-26_F, ~190 kb; pJN2-26 (phage-plasmid), 109.952 kb. The brighter band in JN1a (F-type and HI-type replicons) suggests a larger HI plasmid that runs with the F plasmid, that could not be assembled.

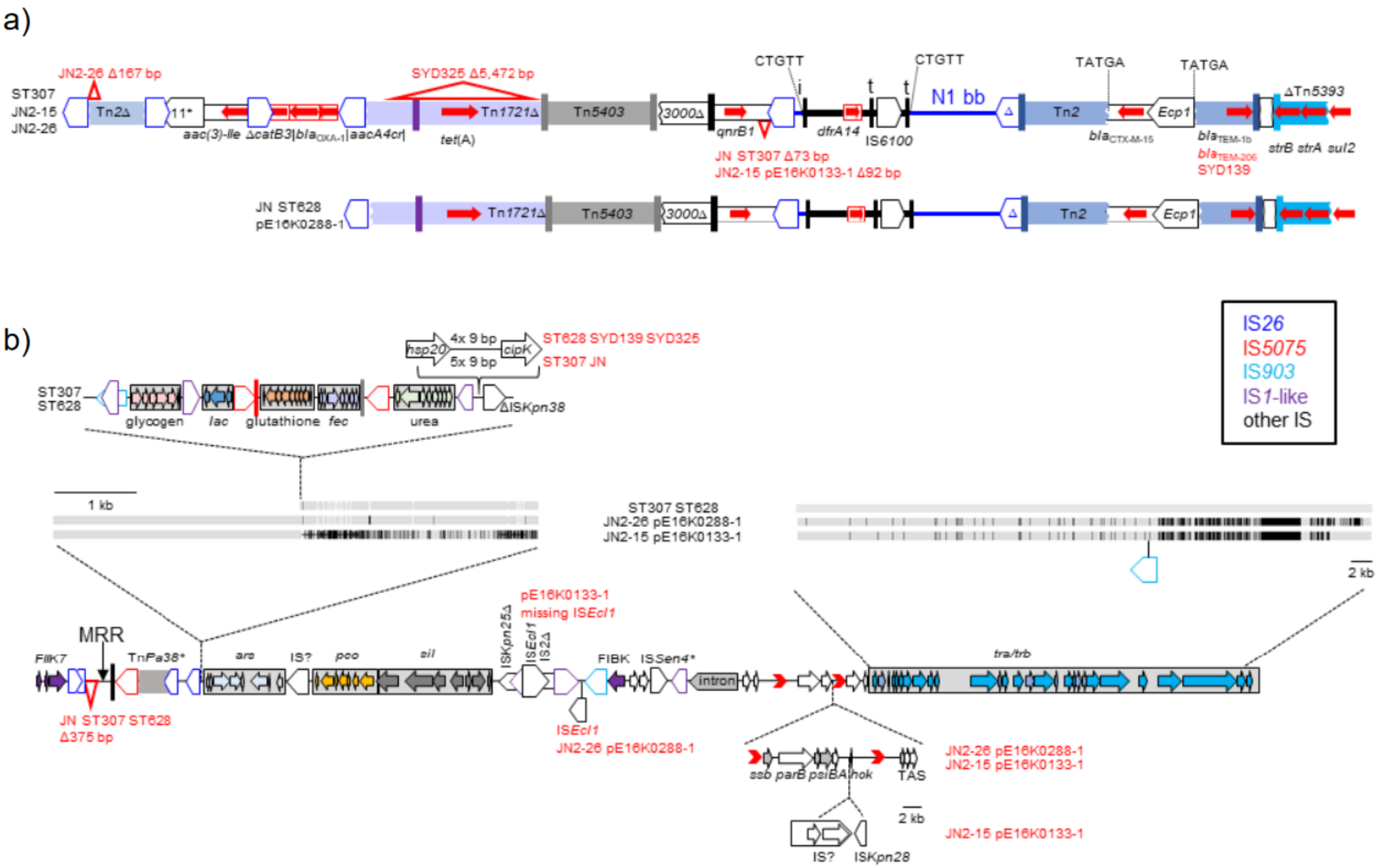

Figure S2. Comparison of FIIK7-FIBK plasmids in different *K. pneumoniae* sequence types. a) Proposed MRR in FIIK7-FIBK plasmids (shown to scale). Resistance genes are shown as labelled red arrows. Red boxes are gene cassettes. Selected insertion sequences (IS) are shown as block arrows (pointed end, right end as defined from direction of transcription of the internal transposase gene), as indicated in the key or labelled. IS*26* flanked regions are shown in an arbitrary order and ST628 plasmids lack one IS*26*-flanked region. Differences between plasmids

are indicated in red. Transposons (Tn) and Tn fragments are shown by wide, coloured lines, with vertical lines representing terminal inverted repeats (IR). i and t indicate IRi and IRt of a class 1 integron in a fragment of N1-type plasmid backbone (N1 bb, blue line) with direct repeats (DR; CTGTT). TATGA, DR flanking an IS*Ecp1*-*bla*$_{CTX\text{-}M\text{-}15}$ transposition unit inserted in Tn*2*. **b)** Comparison of backbones. Components are shown as in part (a), with arrows showing selected genes (to scale in boxed regions only) and *rep* genes in purple. The position of the MRR is indicated by a vertical arrow. JN ST307/ST628 plasmids have three confirmed SNP differences over the whole backbone. They all have five proposed virulence regions (top left) but differ in the number of 9 bp repeats (shown above). pJN2-15_F and JN2-26_F have shorter regions in this position, which differ from each other as shown in the alignment below (grey identical, black different). These plasmids also differ from ST307/ST628 plasmids in the conjugation region (*tra* genes, cyan; *trb* genes, pale blue), shown in the alignment upper right, have additional regions separated by ~450 bp repeats (red chevrons) and extra IS, as indicated.

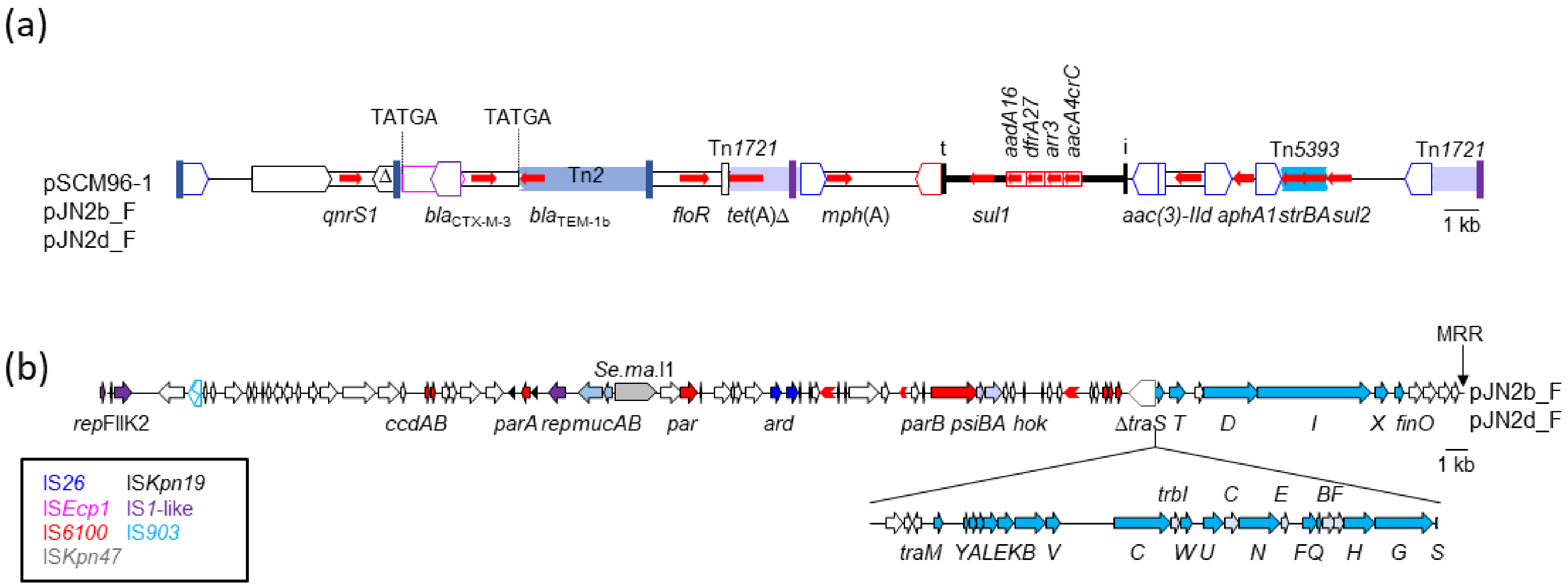

Figure S3. FIIK2-FIB-type plasmid in *K. pneumoniae* ST893 isolates. (a) MRR of pSCM96-1 (CP028717; to scale, as indicated). Antibiotic resistance genes are shown by labelled red arrows. Red boxes are genes cassettes. IS are coloured, as shown in the key. Transposon (Tn) fragments are labelled. Vertical bars represent class 1 integron inverted repeats (IR_i labelled “i”, IRt, “t”) or transposon IR. Contig mapping and assembly graphs of JN2b and JN2d are compatible with the same region being present. **(b)** Backbone of pSCM96-1 (to scale, as indicated), with selected genes labelled. A vertical arrow indicates the position of the MRR. The 26.178 kb segment adjacent to IS*Kpn47* shown below and containing *tra* (cyan) and *trb* (pale blue) conjugation genes, is missing from pJN2b_F/pJN2d_F.

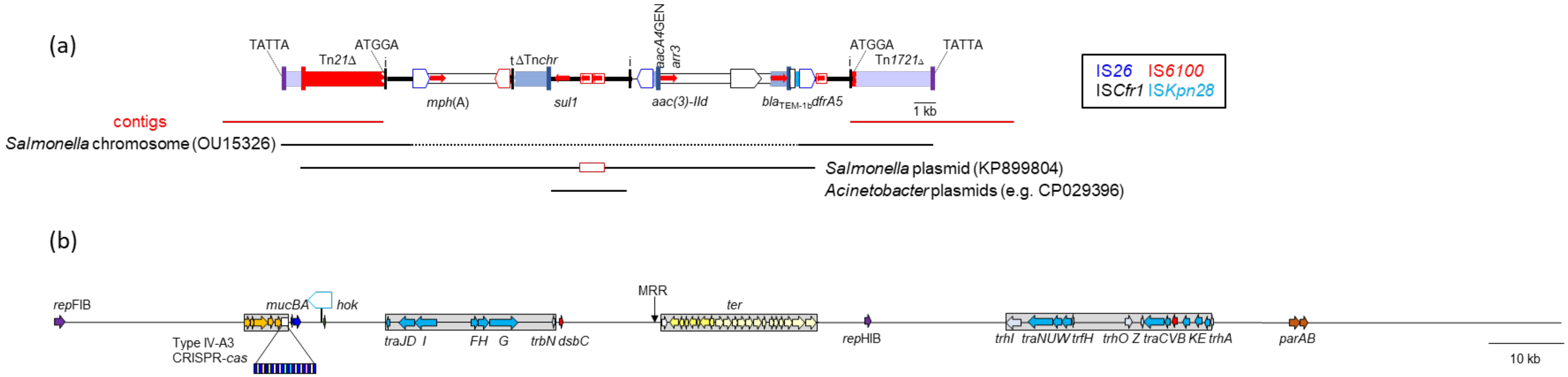

Figure S4. HIB-FIB-type plasmid in *K. pneumoniae* isolates. (a) Proposed arrangement of MRR contigs (to scale, as indicated). Antibiotic resistance genes are shown by labelled red arrows. Red boxes are gene cassettes. IS are coloured, as shown in the key. Transposon (Tn) fragments are labelled. Vertical bars represent class 1 integron inverted repeats (i, IR_i) or transposon IR (t, IRt). The 5 bp sequences are direct repeats flanking transposon or integron IR. Horizontal red lines represent the ends of contigs linking the MRR to the backbone, present in some JN isolates. Horizontal black lines show the extents of regions related to parts of this MRR, with sources and GenBank accession nos. given on the left. In OU15326 itself the regions indicated by solid lines are directly abutted. The red box in KP899804 represents a different cassette array, which can be exchanged between integrons. **(b)** Backbone of HIB-FIB-type plasmid showing selected genes of known function, namely *rep* genes (purple), the Type IV CRISPR-*cas* (orange; with the spacer array expanded below), *mucBA* (blue, *uv* resistance), *hok* (toxin-antitoxin system), *tra* (cyan) and *trh* (pale blue) conjugation genes in two regions, the *ter* region (yellow), *dsbC* genes (red) and *par* genes (brown, partitioning).

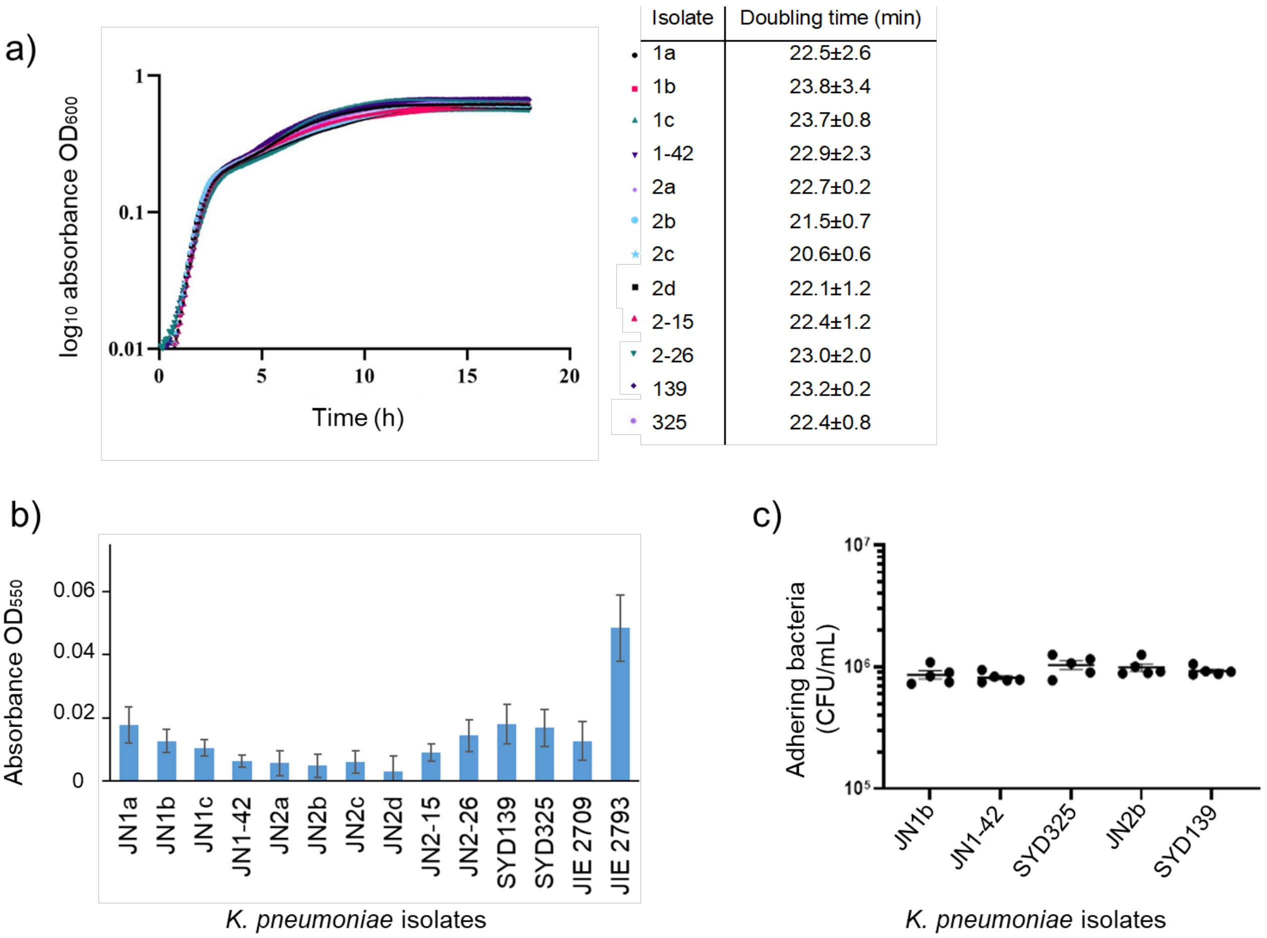

Isolate	Doubling time (min)
1a	22.5±2.6
1b	23.8±3.4
1c	23.7±0.8
1-42	22.9±2.3
2a	22.7±0.2
2b	21.5±0.7
2c	20.6±0.6
2d	22.1±1.2
2-15	22.4±1.2
2-26	23.0±2.0
139	23.2±0.2
325	22.4±0.8

Figure S5. Phenotypic analysis of *K. pneumoniae* isolates. a) Bacterial growth in LB broth measured as absorbance (OD_{600}). Results are expressed as means (± standard errors) for triplicate measurements. Doubling times were calculated on OD_{600} values between 0.02–0.09. **b)** Biofilm production assays in TSB. Results are expressed as means (± standard errors) for two repeated measurements. All isolates were poor biofilm producers in the tested conditions, at similar levels to *K. pneumoniae* ST258 JIE2709 (19). *K. pneumoniae* ST258 JIE2793, a moderate producer (19), was used as positive control. **c)** Adhesion to epithelial cells. ST307, ST628 and ST893 isolates adhere efficiently to human bladder cells. Adherent bacteria were quantified by spot plating on LB agar. Data represent five independent experiments of duplicated assays with standard error mean shown.
